# Retraction: Adsorption of Mn(ii) ions from wastewater using an AgNPs/GO/chitosan nanocomposite material

**DOI:** 10.1039/d6ra90110g

**Published:** 2026-08-21

**Authors:** Abeer El Shahawy, Mahmoud F. Mubarak, Merna El Shafie, Hesham M. Abdulla

**Affiliations:** a Department of Civil Engineering, Faculty of Engineering, Suez Canal University PO Box 41522 Ismailia Egypt abeer_shahawi@eng.suez.edu.eg mernaelshafie24@gmail.com; b Petroleum Applications Department, Egyptian Petroleum Research Institute (EPRI) Nasr City 11727 Cairo Egypt fathy8753@epri.sci.eg; c Faculty of Science, Mansoura University Mansoura Egypt; d Botany Dept., Faculty of Science, Suez Canal University Box 41522 Ismailia Egypt hesham_abdulla@science.suez.edu.eg

## Abstract

Retraction of ‘Adsorption of Mn(ii) ions from wastewater using an AgNPs/GO/chitosan nanocomposite material’ by Abeer El Shahawy *et al.*, *RSC Adv.*, 2022, **12**, 29385–29398, https://doi.org/10.1039/D2RA04693H.

The Royal Society of Chemistry hereby wholly retracts this *RSC Advances* article due to concerns with the reliability of the data.

Fig. 2–4 were duplicated from a previous publication from the same authors without due citation.^1^ In addition, concerns were raised with the integrity of the FTIR data itself in Fig. 2, and the authors have not been able to provide the raw data.

Given the significance of these concerns, the Editor has lost confidence that the findings presented in this paper are reliable.

Hesham Abdulla clarifies that their role was limited to supervising the wastewater analysis conducted at the Suez Canal University Center for Environmental Studies and Consultation, and they were not responsible for generating, processing, validating, or retaining the material-characterization data that are the subject of the concerns raised by the journal, including the FTIR results and Fig. 2–4.

The authors were informed about the retraction of the article but have not indicated whether they agree with the decision to retract.

Laura Fisher

13^th^ August 2026

Executive Editor, *RSC Advances*

**References**

1 A. El Shahawy, M. F. Mubarak, M. El Shafie and H. M. Abdulla, *RSC Adv.*, 2022, **12**, 17065–17084.

